# The diffusion of normal skin wound myofibroblast‐derived microvesicles differs according to matrix composition

**DOI:** 10.1002/jex2.131

**Published:** 2023-12-27

**Authors:** Syrine Arif, Sébastien Larochelle, Benjamin Trudel, Céline Gounou, François Bordeleau, Alain R. Brisson, Véronique J. Moulin

**Affiliations:** ^1^ Faculté de Médecine Université Laval Quebec Quebec City Canada; ^2^ Centre de Recherche du CHU de Québec‐Université Laval Quebec Quebec City Canada; ^3^ Centre de Recherche en Organogénèse Expérimentale de l'Université Laval/LOEX Quebec Quebec City Canada; ^4^ Centre de Recherche sur le Cancer de l'Université Laval Quebec Quebec City Canada; ^5^ UMR‐CBMN CNRS‐Université de Bordeaux‐IPB Pessac France; ^6^ Department of Surgery, Faculty of Medicine Université Laval Quebec City Canada

**Keywords:** alpha2beta1 integrin, diffusion, extracellular vesicles, hydrogel, microvesicles, type I collagen

## Abstract

Microvesicles (MVs) are a subtype of extracellular vesicles that can transfer biological information over long distances, affecting normal and pathological processes including skin wound healing. However, the diffusion of MVs into tissues can be impeded by the extracellular matrix (ECM). We investigated the diffusion of dermal wound myofibroblast‐derived MVs into the ECM by using hydrogels composed of different ECM molecules such as fibrin, type III collagen and type I collagen that are present during the healing process. Fluorescent MVs mixed with hydrogels were employed to detect MV diffusion using fluorometric methods. Our results showed that MVs specifically bound type I collagen and diffused freely out of fibrin and type III collagen. Further analysis using flow cytometry and specific inhibitors revealed that MVs bind to type I collagen via the α2β1 integrin. These data demonstrate that MV transport depends on the composition of the wound environment.

## Graphical abstract

Graphical abstract:

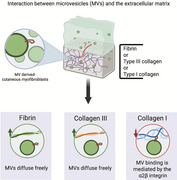

## 1 INTRODUCTION

Skin wound healing has long been a topic of considerable interest in the medical field. It is a complex biological process that involves the coordinated efforts of multiple cell types and extracellular matrix (ECM) components. Effective wound healing is essential for tissue integrity and preventing infection (Singer & Clark, 1999). This highly complex process is made up of four distinct stages (Singer & Clark, 1999), beginning with inflammation and haemostasis followed by proliferation, remodelling and a maturation phase. Among the cells involved, specialized cells known as myofibroblasts (Wmyo) play a significant role in the proliferation stage, where they differentiate from fibroblasts (Arif et al., 2021) and produce microvesicles (MVs) (Moulin et al., 2010). These MVs, a subtype of extracellular vesicles (EVs), are known to promote angiogenesis (Merjaneh et al., 2017) and tissue remodelling (Arif et al., 2020) in vitro.

These EVs are defined as small membrane vesicles released from cells into the extracellular environment. They are known to transport a wide range of molecules such as proteins, lipids and nucleic acids to other cells (Laberge et al., 2018; Narauskaitė et al., 2021; Théry et al., 2018; Yáñez‐Mó et al., 2015). In recent years, much attention has been paid to the study of EVs and their role in cell‐cell communication in the context of wound healing (Joorabloo & Liu, 2023; Laberge et al., 2018; Lu et al., 2022; Narauskaitė et al., 2021). For example, platelet‐derived EVs are involved in regulating haemostasis (Arraud et al., 2014), neutrophil‐derived EVs can exert both anti‐ and pro‐inflammatory functions(Kolonics et al., 2021), endothelial cell‐derived EVs can promote proliferation(Wei et al., 2020) and fibroblast‐derived EVs can contribute to the remodelling phase (Oh et al., 2021).

However, little is known about the mechanisms of MV diffusion within tissues. It is also unclear how the ECM composition might affect this diffusion. During the wound healing process Wmyo interact with the ECM, which undergoes changes through the process. This includes the presence of a temporary matrix called ‘granulation tissue’ that is essentially composed of type III collagen and fibrin, which are present during the proliferation stage. The granulation tissue is eventually replaced by type I collagen produced by resident cells at the end of wound healing (Arif et al., 2021; Diller & Tabor, 2022; Singer & Clark, 1999; Xue & Jackson, 2015). MV diffusion can be influenced by various factors such as the size and shape of the MVs, the properties and composition of the ECM, and the interactions between the MVs and the ECM (Al Halawani et al., 2022; Buzás et al., 2018; Karampoga et al., 2021; Patel et al., 2023) *via* membrane proteins for example (Rieu et al., 2000). Thus, the ECM may impede MV diffusion and affect their role in wound healing.

In this study, we aim to investigate the transport of MVs produced by Wmyo through hydrogels made up of different ECM components present during the healing process. We aim to determine which factors play a role in facilitating or hindering their diffusion. We will specifically examine the effects of the ECM composition on MV behaviour. Our findings suggest that the composition of the ECM plays a significant role in determining the rate of diffusion of MVs. By shedding light on the mechanisms of MV transport through the ECM, our study will contribute to a better understanding of the mechanisms of wound healing and facilitate the development of new therapeutic strategies for tissue repair.

## 2 MATERIAL AND METHODS

### 2.1 Ethical clearance statement

Primary Wmyo were isolated from the wounded skin of adult donors aged 20–40 years. All donors provided their informed consent in writing. Myofibroblasts were obtained from normal human granulation tissue, following the established protocol outlined in prior studies (Germain et al., 1994; Moulin et al., 1999, 2004). Briefly, subcutaneous implantation of polyvinyl alcohol sponges within perforated silicone tubes was performed on volunteer subject's arms. After a 12‐day incubation period, the sponges were recovered, and the cells were extracted using collagenase H and the explant technique. These Wmyo cells, derived from the experimental granulation tissues, consistently exhibited a myofibroblastic phenotype for at least up to the 10th passage in vitro (Moulin et al., 1999).

Protocols were approved by the institutional ethical review board of the CHU de Québec‐Université Laval Research Centre and performed in accordance with the Helsinki Declaration of 1975, as revised in 2008.

### 2.2 Culture of myofibroblast cells

Cells were grown in a culture medium composed of Dulbecco's Modified Eagle's medium (DME, Corning, Manassas, VA, USA) and supplemented with 20% fetal bovine serum (FBS, FB essence, Seradigm, Radnor, PA, USA), 100 U/mL penicillin G (Sigma‐Aldrich, Saint‐Louis, MO, USA) and 25 μg/mL gentamicin (Schering Inc., Kenilworth, New Jersey, USA). Wmyo cells were grown at 37°C in a humidified incubator with 8% CO_2_ and were used at passages between 3 and 8.

### 2.3 Generation of cells expressing mNeonGreen

To obtain cell populations producing fluorescent MVs, three different populations of Wmyo were transduced with the fluorescent protein mNeonGreen. To do so, a gene coding for mNeonGreen‐UtrCH (a gift from Dorus Gadella, Addgene plasmid #98879; http://n2t.net/addgene:98879; RRID: Addgene_98879) (Chertkova et al., 2017) was amplified from the vector using a polymerase chain reaction. The sequence of the forward primer was 5′‐TAAGCAGGGCCCCTACTTGTACAGCTCGTCCAT‐3′ and the reverse primer was 5′‐GTGTATCATATGCCAAGTACGCCCCCTATTGAC‐3′. The amplified fragments were then digested with ApaI and NdeI and ligated in a modified pLenti6.3/V5 plasmid (Thermo Scientific) (Mayrand et al., 2012; Merjaneh et al., 2017) construct. The obtained construct was confirmed as containing the appropriate mNeonGreen‐UtrCH gene using DNA sequencing analysis. For DNA transfection, HEK293T cells were purchased from ATCC (American Type Culture Collection, Manassas, Virginia, USA) and grown in DME supplemented with 10% FBS, 100 U/mL penicillin G and 25 μg/mL gentamicin. The culture was maintained at 37°C in a humid incubator with 5% CO_2_ and passaged every two days. The plasmid containing mNeonGreen was transfected into the cells according to the manufacturer's instructions; Transfection was performed with lipofectamine 2000, OptiMEM and 2 μg/μL of plasmid DNA. 24 h after transfection, the medium was changed, and the virus‐containing supernatants were collected after 48 h. The collected viruses were then diluted in complete DME with 5 mg/mL polybrene and used for the transduction of Wmyo cells. Cells were transduced for 6 h with the third generation self‐inactivating mNeonGreen‐encoding lentivector at a MOI of 3. Following the transduction, positive mNeon‐Green Wmyo cells were sorted using a BD FACSMelody cell sorter (BD Life Sciences, San Jose, CA, USA) with the sorting option and cultured as previously explained (Mayrand et al., 2012; Merjaneh et al., 2017).

### 2.4 Isolation of myofibroblast‐derived microvesicles

MVs were isolated by differential centrifugation as previously described (Arif et al., 2020; Merjaneh et al., 2017). Briefly, when the confluence of Wmyo cells reached 80%, the medium was replaced with DME+20% vesicle‐free FBS supplemented with antibiotics. After 48 h, the conditioned medium was collected and centrifuged for 10 min at 300 × *g* at 4°C. The supernatant of the centrifuged mixture was then centrifuged for 30 min at 21,000 × *g* at 4°C. Finally, the pellet was washed three times with phosphate buffer saline and further centrifuged for 20 min at 21,000 × *g* before experiments.

### 2.5 Spectrophotometry for microvesicle dosage

The protein concentration in MV samples was assessed at 280 nm using a NanoDrop 1000 spectrophotometer (Thermo Fisher Scientific, Mississauga, ON, Canada).

### 2.6 Transmission electron microscopy

As previously described (Arif et al., 2020), the isolated MVs were fixed overnight at 4°C in 2.5% glutaraldehyde (Canemco, Lakefield, QC, Canada) and then stored in a 0.1 M cacodylate buffer (Mecalab, Montreal, QC, Canada). Samples were then stained with 3% uranyl acetate (Sigma, Oakville, QC, Canada) and processed for observation by transmission electron microscopy (TEM) (80 kV, JEOL® electron microscope 1230, Akishima, Tokyo; Institut de biologie intégrative et des systèmes, Microscopy Platform at Université Laval, Quebec City, QC). Multiple pictures were taken at multiple spots. Three samples of MVs isolated from three different Wmyo populations were used for observations. MV size was assessed using ImageJ™ software (https://imagej.nih.gov/ij/).

### 2.7 Cryo‐electron microscopy

Isolated MVs were not subjected to prior fixation or put in a particular buffer before being sent to Cryo‐electron microscopy (Cryo‐EM) (Brisson et al., 2017; Zonneveld et al., 2014). Briefly, a 4 μL aliquot was deposited on an EM grid coated with a perforated carbon film. After draining of the excess liquid with filter paper, the grids were quickly plunged into liquid ethane cooled by liquid nitrogen, using a Leica EMCPC cryo‐chamber. Grids were stored in cryo‐boxes in liquid nitrogen until use. For cryo‐EM observation, grids were mounted onto a Gatan 626 cryoholder and transferred to a Tecnai F20 microscope (Thermo Fisher, USA) operated at 200 kV. Images were recorded with an Eagle 2k CCD camera (Thermo Fisher, USA).

### 2.8 Flow cytometry

Myofibroblast‐derived microvesicles were labelled with either AnnexinV‐Cy5 (AnxV‐Cy5) (Arraud et al., 2015), anti‐CD81‐mAb‐PE (clone JS64, Beckman‐Coulter) or anti‐CD63‐mAb‐PE (clone H5C6, BioLegend) as follows. Microvesicle samples were diluted 300× in a Hepes saline buffer (10 mM Hepes, 150 mM NaCl, 2 mM NaN3, pH 7.4), and added with either 20 ng/mL Anx5‐Cy5, anti‐CD81‐mAb‐PE (final dilution 250x) or anti‐CD63‐mAb‐PE (final dilution 500×) and incubated for 2 h at room temperature before flow cytometry(Arraud et al., 2016). Flow cytometry measurements were performed with a Gallios flow cytometer (Beckman Coulter), with triggering based on fluorescence intensity with settings as described in (Arraud et al., 2015).

### 2.9 Hydrogel preparation

Type I collagen hydrogel was produced using PureCol® EZ Gel (Bovine Collagen, Solution, Advanced BioMatrix, Inc, Carlsbad, CA, USA). The gel was mixed with a solution composed of NaCl (0.9%), CaCl^2+^ (1 mM), and Mg^2+^ (1.4 mM) to a final concentration of 2.5 or 4 mg/mL of collagen. Type III collagen hydrogel was prepared with a solution composed of 70% human type III collagen (Advanced BioMatrix, Inc) at a final concentration of 4 mg/mL mixed with 30% type I collagen at 4 mg/mL. For the fibrin hydrogel, thrombin (Sigma) diluted in a solution composed of NaCl (0.9%) with 0.1% BSA was added to a fibrinogen solution (Sigma) diluted in 0.9% NaCl (final concentration of fibrinogen 2 mg/mL, thrombin 1 U/mL). For all solutions, the pH of the mix before polymerization was adjusted to 7. Hydrogels were either premixed with 40 mg of fluorescent MV proteins diluted in NaCl+CaCl^2+^/Mg^2+^ buffer solution or with buffer only, and left to polymerize for a minimum of 3 h at 37°C before adding the same volume of NaCl+CaCl^2+^/Mg^2+^ buffer on the top of the hydrogels. Another variation of the experiment was carried out by first letting the hydrogel polymerize and then adding the NaCl+CaCl^2+^/Mg^2+^ buffer containing MVs on top of the hydrogel. To establish a baseline for MV detection, a control was conducted using a well containing only MVs diluted in the buffer without hydrogel.

### 2.10 Microvesicle release detection and pore size analysis

The release of MVs from hydrogels after 24 h was assessed by collecting the buffer that was added on top of the hydrogels following polymerization. In experiments that required more than 24 h observation, a fresh buffer containing no MVs was substituted on top of the hydrogel. MV fluorescence was detected by microplate reader (Varioskan Flash, ThermoFisher Scientific) at the fluorescence Exλ/Emλ set at 506/517 nm. The microplate reader generated raw data expressed as Relative Fluorescence Units (RFU). To enhance data accuracy, a correction was applied by subtracting the corresponding blank value from each condition. For instance, when analysing a condition with type I collagen and MVs, the RFU of a blank well containing only type I collagen without MVs was subtracted. The volume of liquid added atop the hydrogel matched the hydrogel's volume. Upon MV addition to the hydrogel, in the absence of binding between MVs and hydrogel, they freely diffused out to achieve equilibrium, reaching a concentration equal to half that at *t* = 0 within the hydrogel. Consequently, fluorescence of an internal control, representing half the MVs amount added to hydrogels, was measured. The corrected data were transformed into percentages using the internal control as a reference for each experiment to depict the extent of MV release from the hydrogel samples.

### 2.11 Confocal reflectance microscopy

Collagen pore size was computed using the autocorrelation method as before (Bordeleau et al., 2013) with the following modifications: Freshly prepared type I collagen was imaged with confocal reflectance microscopy. The fibre structure of collagen gels was visualised using a Zeiss LSM 900 inverted laser scanning microscope (Carl Zeiss Microscopy USA, Thornwood, NY) equipped with a 644 nm laser and a 40×/1.1 NA water‐immersion objective (Carl Zeiss). Images were acquired with a pinhole size optimised to provide a 1 μm optical section.

All the images were preprocessed using the OpenCV (Open Source Computer Vision Library) package for Python. Briefly, noise reduction through a median filter of 5 × 5 pixels was applied and then the intensity was adjusted according to the local maximum with a kernel of size 31 × 31 pixels. A threshold based on the ‘triangle’ algorithm was then applied to the image followed by a 3 × 3 pixel Gaussian smoothing filter. The autocorrelation function was computed using MATLAB and then the images were transferred back into Python. The resulting autocorrelation signal was fitted to a gaussian surface. The effective pore size diameter was computed based on the 1/e^2 value of the fitted gaussian curve.

### 2.12 Microvesicle surface protein pretreatments

Extravesicular domains of proteins on MVs were cleaved by proteinase K digestion at 20 μg/mL (PB0451, BioBasic, Canada) diluted in NaCl+CaCl^2+^/Mg^2+^ buffer and incubated for 1 h at 37°C following Mikhail Skliar's method (Skliar et al., 2018). Proteinase K activity was then terminated by heating the sample to 65°C for 10 min. MVs were then centrifuged at 20,000 × *g* for 20 min to remove any remaining debris. For RGD peptide treatments, MVs were incubated with either RGD peptide (H‐7630.0005, BACHEM, USA) or its control RGE peptide (H‐3136.0005, BACHEM) at 100 μg/mL in NaCl+CaCl^2+^/Mg^2+^ buffer for 2 h at 37°C followed by a wash at 20,000 × *g* for 20 min to remove any remaining debris. BTT‐3033 treatment (4724, TOCRIS, Biotechne) was used to selectively inhibit α2β1 integrin. MVs were incubated with 10 μM/mL of BTT‐3033 in NaCl+CaCl^2+^/Mg^2+^ buffer for 1 h at 30°C and then centrifuged at 20,000 × *g* for 20 min to remove any remaining debris before to be added in hydrogels.

### 2.13 Detection of integrins

The immunolabelling of fluorescent MVs was performed with α2β1 antibody (10 μg/mL, MAB1998, Millipore, USA), α1 antibody (IgG1 mouse anti human antibody generated by our research centre) or with an isotype control IgG1 (10 μg/mL, negative control mouse IgG1, X0931, Dako, Denmark) followed by phycoerythrin‐conjugated goat anti‐mouse IgG1 antibody (1:10, P21129, Invitrogen, USA). Following their acquisition by flow cytometry (Becton Dickinson, Mississauga, ON, Canada), mNeonGreen‐MVs were first gated and the fluorescence of antibodies was analysed using Cyflogic 1.2.1 Software (https://www.cyflogic.com/).

### 2.14 Statistical analysis

All statistical analysis was performed using GraphPad Prism (GraphPad Software Inc., version 9.4.0). All experiments were performed in triplicates to ensure biological reproducibility. Statistical significance was defined as *p* < 0.05 and was determined for each experiment as described in the corresponding figure legend.

## 3 RESULTS

### 3.1 Characterisation of microvesicles produced by myofibroblasts

**FIGURE 1:**
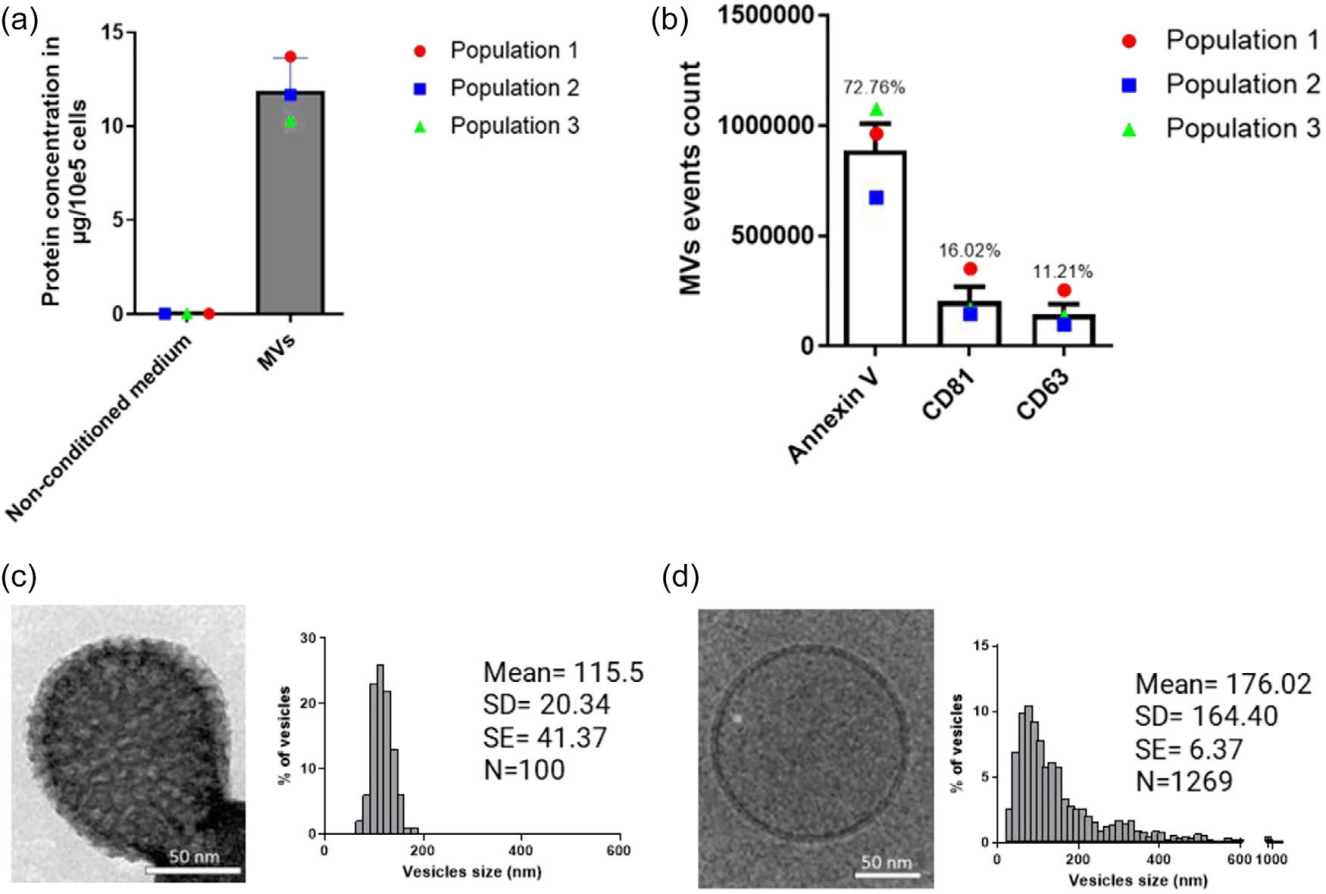
Characterization of microvesicles produced by myofibroblasts. (a) MVs samples were quantified by measuring protein concentration using spectrophotometric analysis and reported to the number of producing cells. (b) Relative distribution of MV populations expressing either phosphatidylserine (AnxV‐labeled), CD81 or CD63, determined by flow cytometry. % for each marker are indicated above the respective columns. The data are represented in terms of the mean value  ±  the standard deviation (SD) over triplicates of three groups of MVs isolated from three different populations of Wmyo (*N* = 3, *n* = 3). Each dot represents the mean for each population used in this experiment. The size and morphology of isolated MVs were determined by several methods: (c) Representative TEM picture of MVs produced by Wmyo and core size distribution. (d) Representative cryo‐EM picture of MVs produced by Wmyo and core size distribution.

MVs were isolated from Wmyo culture supernatant and subjected to various analyses to characterise them. It should be noted that MVs have previously been characterised in our lab (Arif et al., 2020; Merjaneh et al., 2017; Moulin et al., 2010). The protein concentration of the MVs was quantified using a NanoDrop spectrophotometer. Our observations showed the presence of 11.89 ± 1.41 μg MV protein/10^5^ cells for the three different samples of MVs isolated from three different Wmyo populations (Figure 1a). The presence of EV markers was evaluated by conducting flow cytometry in the presence of specific antibodies. The majority of MVs (72.76% ± 7.45) were positively labelled with Annexin V. By contrast, a small percentage of MVs tested positive for CD81 (16.02% ± 4.47) or for CD63 (11.21% ± 3.05), two markers primarily associated with exosomes (Figure 1b). The size and morphology of isolated MVs were determined by imaging methods. According to TEM and cryo‐EM images, MVs have a spherical shape and a noticeable lipid bilayer (Figure 1c,d). The size range of MVs was 115.50 ± 20.34 nm as determined by TEM analysis (Figure 1c), and 176.02 ± 164.40 nm as determined by cryo‐EM analysis (Figure 1d) for three different samples of MVs derived from three different Wmyo populations. Furthermore, our earlier research has shown that Wmyo‐derived MVs do not include apoptotic bodies (Moulin et al., 2010) and our large‐scale cryo‐EM images reveal a low presence of contaminants (Figure S1). These characterizations collectively support the identification of MVs as a subtype of EVs.

**FIGURE 2:**
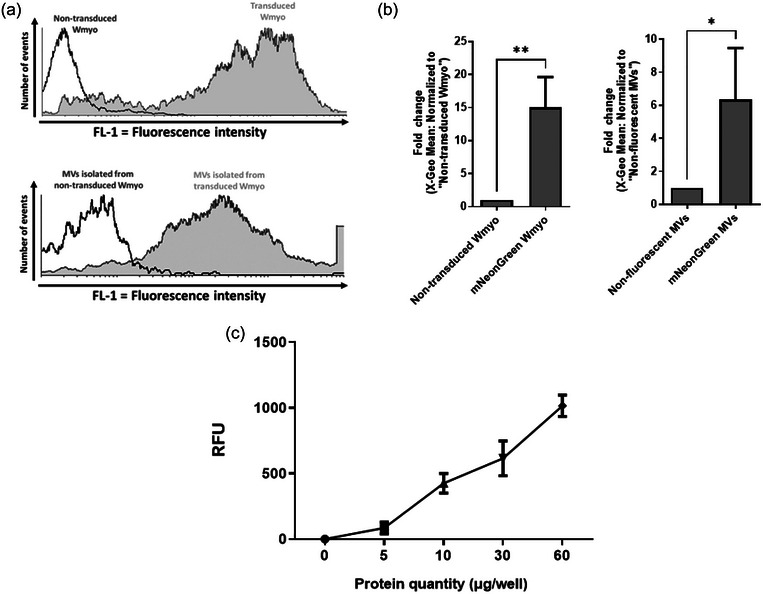
Generating fluorescent microvesicles. Wmyo were first transduced with a fluorescent protein (mNeonGreen) to generate fluorescent MVs. MVs were then isolated from Wmyo culture media using differential centrifugations. Subsequently, fluorescent MVs presence was confirmed by several methods: (a) Representative flow cytometry histograms of transduced Wmyo labelled using the fluorescent protein mNeonGreen (Top histogram) and their produced fluorescent MVs (Bottom histogram) showing the number of events versus the fluorescence intensity. Non‐transduced Wmyo and MVs were used as a control (empty curves). (b) Graphical analysis of pooled data of (a) fluorescent Wmyo and MVs. (c) Relative fluorescence unit (RFU) according to protein quantity of fluorescent MVs. The data are represented in terms of the mean value ±  the standard deviation (SD) over triplicates of three groups of MVs isolated from three different populations of Wmyo (*N* = 3, *n* = 3). Statistical analysis was performed using two‐tailed *T*‐test. Statistical significance was indicated as follows: **p < 0.01 and *p < 0.05.

In order to detect MVs in our experiments, we generated fluorescent cells able to produce fluorescent MVs. To achieve this, Wmyo cells were transduced with a plasmid carrying an UtrCH‐mNeonGreen fusion protein. UtrCH is the calponin homology domain of utrophin, an actin‐binding protein (Winder et al., 1995). Utrophin has been shown to bind actin and to be anchored at the inner membrane leaflet in association with a glycoprotein complex (Khurana et al., 1999; Winder et al., 1995). It has been reported to bind F‐actin without stabilizing it (Burkel et al., 2007; Rybakova & Ervasti, 2005) nor altering the actin cytoskeleton (Burkel et al., 2007). Additionally, utrophin has been reported to be expressed in EVs (Guan et al., 2021). Flow cytometry analysis showed that Wmyo expressed fluorescent protein in quantities about 15.03 ± 3.74 times higher than non‐transduced Wmyo cells (Figure 2a,b). Fluorescence intensity in MVs increased 6.36 ± 2.54 times compared with non‐fluorescent MVs (Figure 2a,b). The presence of MVs was also detectable by the fluorometric approach using a plate reader, and the intensity of the fluorescence increased with the quantity of MVs added to the wells (Figure 2c). Furthermore, proteinase K treatment did not alter fluorescence intensity in MVs, indicating encapsulation of the mNeonGreen fluorescent protein by MVs (Figure S2). These findings indicate that fluorescent MVs can be effectively produced by transduced Wmyo. This makes them easily detectable by some approaches without the addition of markers or external agents.

### 3.2 The diffusion of microvesicles differed according to the type of ECM hydrogel

**FIGURE 3:**
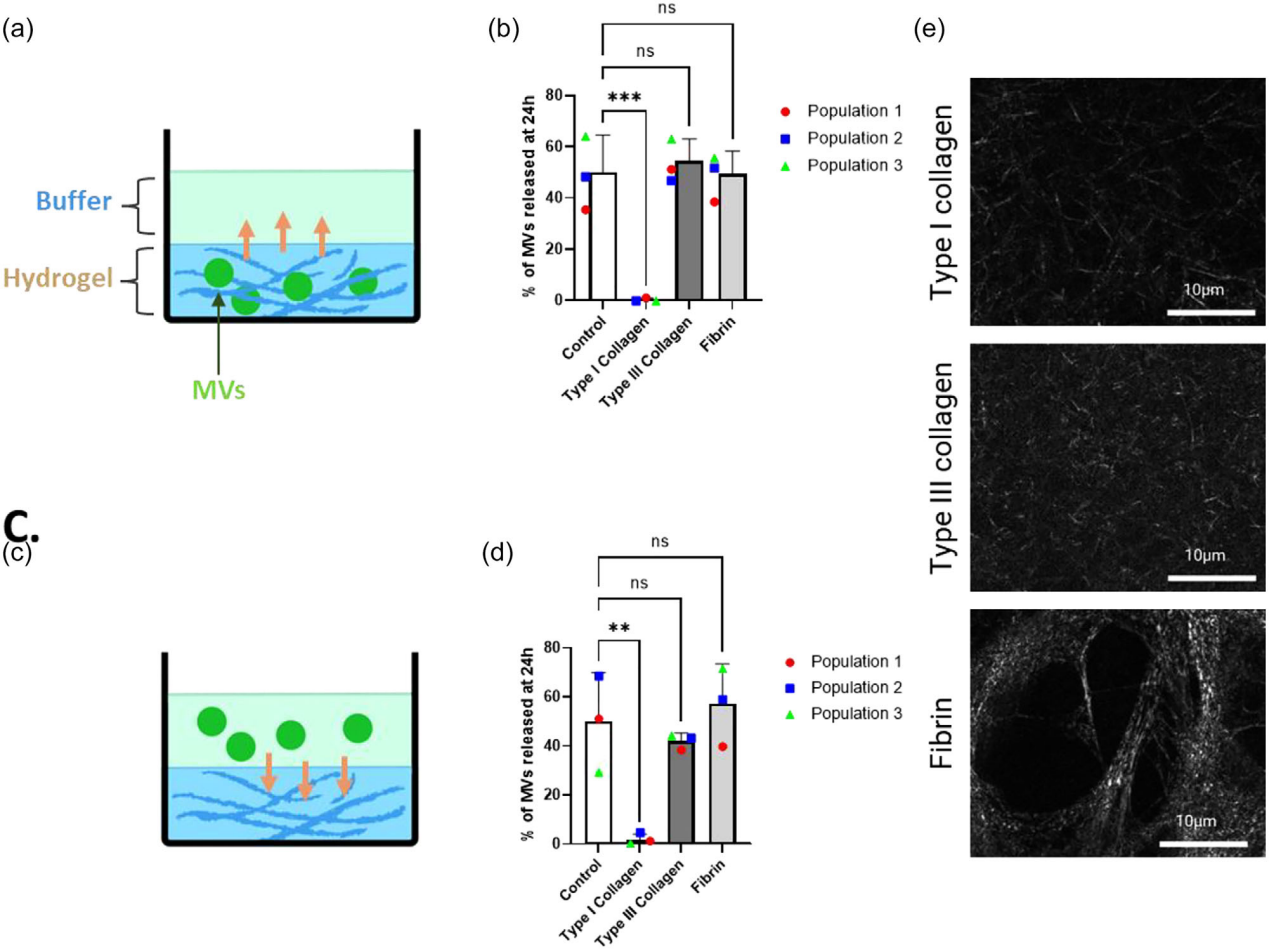
The release profile of MVs from type I collagen, type III collagen and fibrin hydrogels. Release profile of MVs from type I collagen, type III collagen and fibrin hydrogels. (a) Schematic illustration depicting the release of fluorescent MVs pre‐mixed with hydrogels to the buffer. (b) The release profile of MVs following fluorescence in the buffer after 24 h. (c) Schematic illustration depicting the diffusion of fluorescent MVs included in the buffer on top of the readily polymerized hydrogel. (d) Bar graph depicting the presence of MVs after 24 h in the buffer. The data are represented in terms of the mean value  ±  the standard deviation (SD) over triplicates of three groups of MVs isolated from three different populations of Wmyo (*N* = 3, *n* = 3). Each dot represents the mean for each population used in this experiment. Statistical analysis was performed using one‐way ANOVA with Dunnett's multiple comparisons test, with control samples used as the reference. Statistical significance was indicated as follows: ns (not significant), **p < 0.01 and ***p < 0.001. (e) Confocal reflectance microscopy of each type of hydrogel.

To assess the diffusion of MVs into the wound environment, we selected fibrin, type III collagen and type I collagen for inclusion in our test hydrogels. These molecules are the most important proteins in the ECM when Wmyo are present in the wound (Xue & Jackson, 2015). We used hydrogels composed of a single type of ECM molecule as a study model. This allowed us to focus on the specificity of binding of MVs to individual ECM molecules. MVs were first added to hydrogels before their polymerization. A buffer was then added to the top of the hydrogels and recovered after 24 h to evaluate the presence of fluorescent MVs (Figure 3a). When hydrogels were composed of type III collagen or fibrin, MVs freely diffused outside of the hydrogels and the intensities of the fluorescence were similar to the control (where the hydrogel was replaced by the same volume of buffer) (Figure 3b). By contrast, no fluorescence was detected in the buffer of the type I collagen hydrogel after 24 h (Figure 3b). We also tested whether adding MVs to the buffer after hydrogel polymerization alone would affect their diffusion (Figure 3c). The result showed a similar outcome to the first model (Figure 3d). In both cases, about 50% of the MVs could be detected in the buffer when the type III collagen and fibrin hydrogels were used, showing an equilibrium in the amount of MVs between the two compartments. In contrast, in the presence of type I collagen, MVs remained trapped in the hydrogel.

### 3.3 The retention of microvesicles by type I collagen was not limited by steric hindrance

**FIGURE 4:**
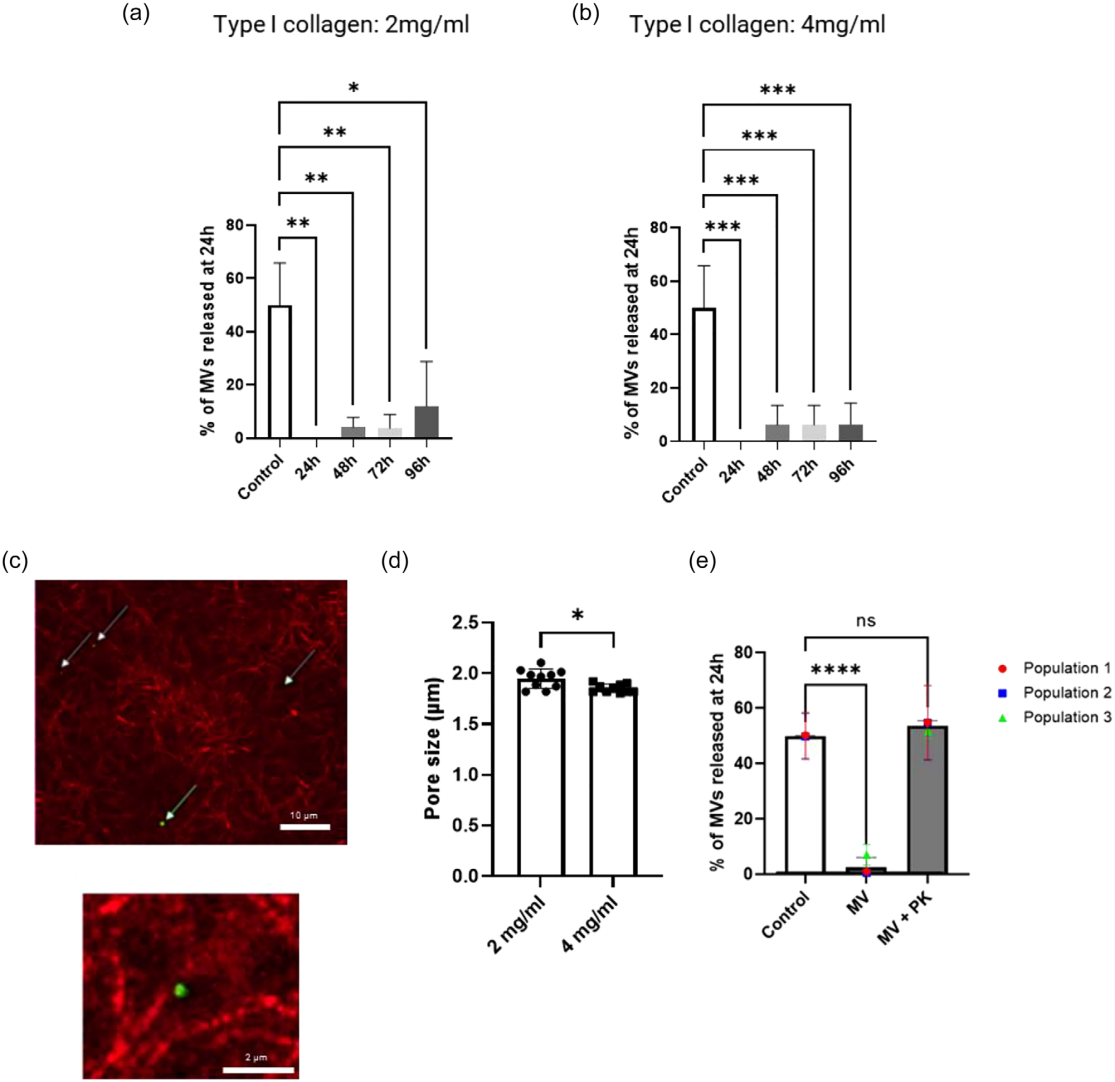
The microvesicles retention by type I collagen was not limited to steric hindrance. (a) The release profile of MVs over 96 h from type I collagen at a concentration of 2 mg/mL, and (b) at a concentration of 4 mg/mL. The pore size of type I collagen hydrogel was then evaluated by confocal reflectance microscopy. (c) Type I collagen hydrogel at 4 mg/mL mixed with fluorescent mNeonGreen MVs (in green, arrows). (d) Evaluation of pore size of type I collagen hydrogel at 2 and 4 mg/mL. (e) Release profile of MVs from type I collagen hydrogels following proteinase K digestion of MVs. For (a) and (b), the data are representative of one population. They are represented in terms of the mean value ±  the standard deviation (SD) over triplicates of one group of MVs isolated from two population of Wmyo (*N* = 2, *n* = 3). For (d), The data are represented in terms of the mean value  ±  the standard deviation (SD) over n = 10 for 3 images. For (e), the data are represented in terms of the mean value ±  the standard deviation (SD) over triplicates of three groups of MVs isolated from three different populations of Wmyo (*N* = 3, *n* = 3). Each dot represents the mean for each population used in this experiment. Statistical analysis was performed using one‐way ANOVA with Dunnett's multiple comparisons test, with control samples used as the reference for (a), (b), and (e). For (d), statistical analysis was performed using Welch's *t*‐test. Statistical significance was indicated as follows: ns (not significant), *p < 0.05, **p < 0.01, ***p < 0.001 and ****p < 0.0001.

The diffusion of MVs through hydrogels is governed by the size of the vesicles and/or their interactions with the hydrogel components (Lieleg & Ribbeck, 2011). One of the characteristics of hydrogels is their pore size, which allows steric filtering of particles according to their size and shape and prevents their passage through the porous medium. The inability of MVs to diffuse out of the type I collagen hydrogel could be caused by the pores being smaller than the size of the MVs. Since the pore size is inversely proportional to the protein concentration of the ECM, two different concentrations of type I collagen (2 and 4 mg/mL) were used to investigate the diffusion of MVs. However, both concentrations gave similar results, as MVs remained trapped within the hydrogel even after a 96‐hour period (Figure 4a,b). Using confocal reflectance, we evaluated the pore size of the type I collagen hydrogel. The pore sizes were found to be 1.85 ± 0.04 μm (Figure 4c,d), 10 times larger than the size of the MVs. Of note, the MVs were observed in close proximity to collagen fibres (in green, Figure 4c). This suggest that MVs may possess membrane molecules/proteins capable of binding to type I collagen.

### 3.4 Microvesicles expressed integrins on their membrane

We then aimed to investigate whether the proximity of MVs to collagen fibres is mediated by membrane proteins. To test this, MVs were pre‐treated with proteinase K, a nonspecific protease that digests the majority of surface proteins on EVs (Skliar et al., 2018). MVs pre‐treated with proteinase K diffused freely out of the type I collagen hydrogels, compared with untreated MVs (Figure 4e) although MV integrity was retained following this treatment (Figure S2). This suggests that membrane proteins would be responsible for the binding of MVs to type I collagen.

**FIGURE 5:**
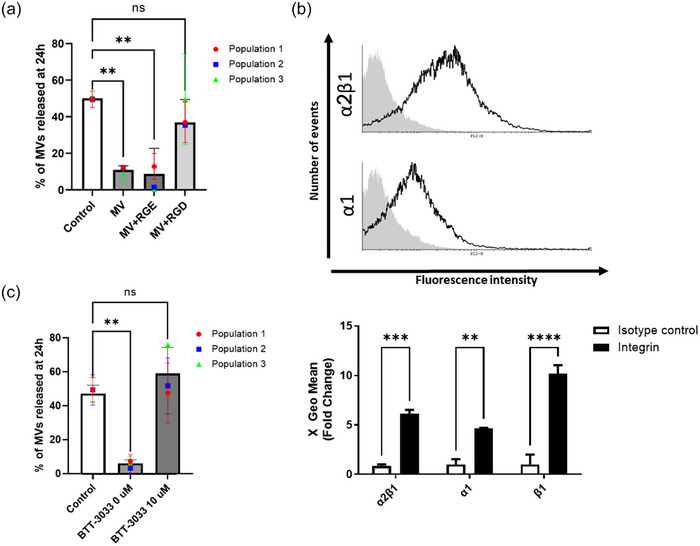
Selective inhibitors disrupt microvesicle binding to type I collagen. (a) Diffusion of MVs outside type I collagen hydrogels when MVs were pre‐treated with RGD peptide or its negative control RGE peptide prior to their incorporation in hydrogel. (b) Flow cytometry detection of surface integrins on MVs. The fluorescence intensity was quantified, and the average of measured geometric means is displayed. Grey filled histograms represent the isotype controls and bold black lined histograms represent the antibody detected. (c) Diffusion of MVs outside type I collagen hydrogels when MVs were pre‐treated with BTT‐3033 prior to their incorporation in the hydrogel. For all figures, the data are represented in terms of the mean value ± the standard deviation (SD) over triplicates of three groups of MVs isolated from three different populations of Wmyo (*N* = 3, *n* = 3). Each dot represents the mean for each population used in this experiment for (a) and (c). Statistical analysis was performed using one‐way ANOVA with Dunnett's multiple comparisons test, with isotype control samples used as the reference for (a) and (c). For (b), statistical analysis was performed using two‐way ANOVA with Bonferroni's multiple comparisons test. Statistical significance was indicated as follows: ns (not significant), **p < 0.01, ***p < 0.001 and ****p < 0.0001.

While several surface proteins can bind to the ECM, integrins are the main receptors that mediate adhesion (Hynes, 1992; Ruoslahti & Pierschbacher, 1987). Most integrins recognise a universal tripeptide sequence containing arginine‐glycine‐aspartic acid (RGD) (Goligorsky et al., 1998; Hynes, 2002; Jokinen et al., 2004; Pierschbacher & Ruoslahti, 1987; Ruoslahti & Pierschbacher, 1987). MVs were pre‐treated with RGD peptide before being added to a type I collagen hydrogel. Treatment with the RGD peptide induced the diffusion of MVs outside the type I collagen hydrogel (Figure 5a), in contrast with its negative control the RGE peptide, a sequence that is not recognised by integrins. This suggests that integrins are likely responsible for the binding of MVs to type I collagen.

### 3.5 Selective inhibitors disrupted microvesicle binding to type I collagen

The integrins most reported to bind type I collagen are α2β1 and α1β1 (Jokinen et al., 2004). We then evaluated the presence of these integrins on the surface of MVs using flow cytometry. Our results show that both α2β1 and α1 were present on both MVs and their parent cells (Figure 5b).

Our investigation centred on α2β1 integrin, given its higher affinity for type I collagen compared with α1β1 integrin (Gullberg et al., 1992; Hynes, 2002; Xu et al., 2000). To selectively inhibit the binding of α2β1 integrin with the ECM, we used a specific inhibitor: the small molecule BTT‐3033 (Salemi et al., 2021). MVs treated with BTT‐3033 freely diffused in the presence of type I collagen hydrogel (Figure 5c). This result indicated that the binding of MVs to type I collagen was facilitated by α2β1 integrin on their surface.

## 4 DISCUSSION

Despite the growing interest in EV biology, the mechanisms underlying their transport through the ECM remain poorly understood. This knowledge gap is a significant obstacle to understanding the extent of EV‐mediated intercellular communication and their role in tissue repair and disease progression.

In contrast to our previous studies, which primarily examined the effects of MVs in a conventional 2D setting using monolayer cultures (Arif et al., 2020; Merjaneh et al., 2017), the present study represents a significant step forward by embracing the complexity of a 3D hydrogel environment, mimicking the in vivo conditions of the wound healing process. In our prior methodologies, cells were first cultured on plastic surfaces, then MVs were introduced to the cells and subsequent effects were assessed. In these studies, MVs revealed their capacity to stimulate angiogenesis in endothelial cells (Merjaneh et al., 2017) and contribute to matrix remodelling by fibroblasts (Arif et al., 2020). However, the transition to a 3D setting introduces a more intricate dynamic, where the diffusion or binding of MVs to the ECM plays a pivotal role. Notably, the skin wound healing process involves a highly dynamic ECM, and the diffusion behaviour of MVs may differ based on the 3D matrix. Unlike the conventional monolayer cultures, our current study emulated the in vivo environment more faithfully, shedding light on the distinct mechanisms influencing MV behaviour during different stages of wound healing.

Additionally, in the existing literature, limited attention has been directed towards the free diffusion of EVs, with most studies primarily focusing on EV binding to specific ECM proteins—a facet we will delve into in this discussion. However, even in these studies, the precise mechanisms underlying EV binding remained largely unexplored. In contrast, our study goes beyond the existing literature by meticulously evaluating the interactions of MVs with the principal ECM components surrounding them.

Despite the importance of the ECM in the in vivo environment, the role of the ECM in EV action has not yet been considered. Therefore, our current study aims to explore MV diffusion in a 3D environment closer to the Wmyo cell environment throughout the wound healing process.

Our findings suggest that MVs can diffuse freely through type III collagen and fibrin, which are the primary components of the temporary ECM that is formed during the early stages of wound healing (Diller & Tabor, 2022; Gurtner et al., 2008; Midwood et al., 2004; Singer & Clark, 1999). This could promote the early steps of wound healing, stimulating fibroblast and endothelial cell migration and growth (Arif et al., 2020; Merjaneh et al., 2017) into the granulation tissue from the border of the wound and thus increasing the number of cells in the wound.

In contrast, MVs stably bound to the type I collagen matrix did not diffuse out of the gel even after a few days. While type I collagen is absent at the beginning of wound healing, this protein increases in quantity with time and becomes the major component of the ECM at the latter steps of the healing process (Xue & Jackson, 2015; Zhang et al., 2023). The lack of diffusion may be attributed to the properties of the ECM or its porosity. Some tissues have an ECM with pore sizes smaller than the size of EVs, which can trap them (Kosto & Deen, 2005; Lenzini et al., 2020; Parlato & Murphy, 2014; Stylianopoulos et al., 2010). Other studies report that EVs appear to bind to ECM fibrils, despite being smaller than the ECM pore size (Zhang et al., 2020). Thus, the passive transport of MVs within the ECM relies on the interplay between MV properties and the ECM network parameters. Our findings, obtained through confocal reflectance microscopy on type I collagen hydrogels, reveal that the pore size is larger than the size of the MVs. In addition, MVs were located in close proximity to collagen fibrils, indicating a potential connection between MVs and type I collagen. MVs were subjected to RGD peptide treatment to investigate the involvement of integrins in MVs binding. In this context, the short three‐amino acid peptide sequence of the RGD peptide bound to MVs integrins. The RGD peptide effectively prevented MV integrins from accessing type I collagen. Treatment of MVs with a specific α2ß1 inhibitor, BTT‐3033, yielded concordant outcomes, supporting the notion that MVs bind to type I collagen via the α2β1 integrin expressed on their extravesicular side. While other integrins can also bind type I collagen, their binding affinity is not as strong as that of α2β1 integrin (Kehrel et al., 1998; Kern et al., 1993; Tulla et al., 2001) and they were not present within our MVs (data not shown). This limited diffusion of MVs outside the granulation tissue could limit the recruitment of cells, such as endothelial cells and restrict neoangiogenesis. The pro‐angiogenic stimulation of MVs is particularly relevant during the early stages of wound healing, but may become less important in later stages of the process (Iruela‐Arispe & Dvorak, 1997). Furthermore, the binding of MVs to type I collagen suggests that MVs may potentially serve as a reservoir of intercellular signals for nearby cells, such as dermal fibroblasts, promoting ECM remodelling as previously suggested (Al Halawani et al., 2022; Patel et al., 2023; Rilla et al., 2019). For example, EVs anchored to the urinary bladder ECM have been shown to suppress pro‐inflammatory signalling (Van Der Merwe et al., 2019; Zhang et al., 2013). Additionally, a recent study demonstrated that EVs isolated from human breast cancer cell lines bind to laminin‐rich ECM (Sariano et al., 2023) which might be in turn useful for cell signalling. Therefore, our study suggests that MVs bound to type I collagen could play a crucial role in the promotion of ECM remodelling and the regulation of angiogenesis during the healing process.

Looking ahead, the insights gained through our findings may pave the way for future clinical applications in the context of EV‐based therapies. Within the evolving biomedical landscape, EVs have surfaced as promising alternatives to cell‐based therapies, distinguished by their heightened biocompatibility, low toxicity, minimal immunogenicity, and inherent potential for engineering (Murphy et al., 2019). However, their clinical application faces challenges such as rapid clearance from the bloodstream and unintended interactions with non‐target cells or tissues (Hallal et al., 2022; Jarmalavičiūtė & Pivoriūnas, 2016; Murphy et al., 2019). While not currently employed in clinical settings, the prospective utility of Wmyo‐derived MVs in specific clinical applications holds promise. Considering the central involvement of Wmyo in fibrotic processes (Hinz et al., 2007; Moulin et al., 1999, 2004), MVs derived from these cells may present opportunities for therapeutic intervention. Moreover, in scenarios of profound skin injuries, such as those experienced by severe burn patients, MVs could potentially contribute to the modulation of inflammatory responses due to the presence of some cytokines of interests on their surface (Arif et al., 2020). However, these hypothetical applications necessitate more extensive investigations, encompassing not only the bioactive effects of MVs but also their intricate interplay with organ systems. Critical areas for exploration include the nuanced diffusion of MVs, particularly through capillaries during wound healing, which may be intricately linked to MVs' dynamic interactions with the diverse ECM components throughout distinct stages of the wound healing process. Rigorous studies addressing these multifaceted aspects are imperative to advance our understanding and pave the way for potential clinical applications of Wmyo‐derived MVs.

Overall, the findings of this study demonstrate the importance of considering the role of MV transport in the ECM in parallel with the direct action of MVs on cells.

## 5 CONCLUSION

The role of MVs in regulating biological processes, including wound healing, is of critical importance. However, progress in this field has been hindered by a limited understanding of the fundamental mechanisms of MV transport in tissues. Our study has contributed to a better understanding of the interactions of Wmyo‐derived MVs and various ECM proteins, which is a novel finding rarely observed in previous studies. A better understanding of EV diffusion through the ECM is crucial in understanding the regulation of intercellular communication, tissue repair and disease progression. Our findings provide new insights into the role of EVs in these processes and could lead to the development of new therapeutic methods targeting EVs.

## AUTHOR CONTRIBUTIONS

**Syrine Arif**: Conceptualization; formal analysis; investigation; methodology; writing—original draft. **Sébastien Larochelle**: Methodology; writing—review and editing. **Benjamin Trudel**: Methodology; writing—review and editing. **Céline Gounou**: Methodology; writing—review and editing. **François Bordeleau**: Methodology; writing—review and editing. **Alain R. Brisson**: Methodology; writing—review and editing. **Véronique J. Moulin**: Conceptualization; data curation; formal analysis; funding acquisition; methodology; project administration; supervision; writing—original draft.

## CONFLICT OF INTEREST STATEMENT

The authors report no conflict of interest.

## Supporting information

Supplementary Information
