# Supplementary material for: The diffusion of normal skin wound myofibroblast‐derived microvesicles differs according to matrix composition
Source: J Extracell Biol. 2023 Dec 27;3(1):e131. doi: 10.1002/jex2.131 (PMC11080821; doi:10.1002/jex2.131)
Supplement: Supplementary file 1 — Supplementary Information [file JEX2-3-e131-s001.docx]

**Supporting Information**


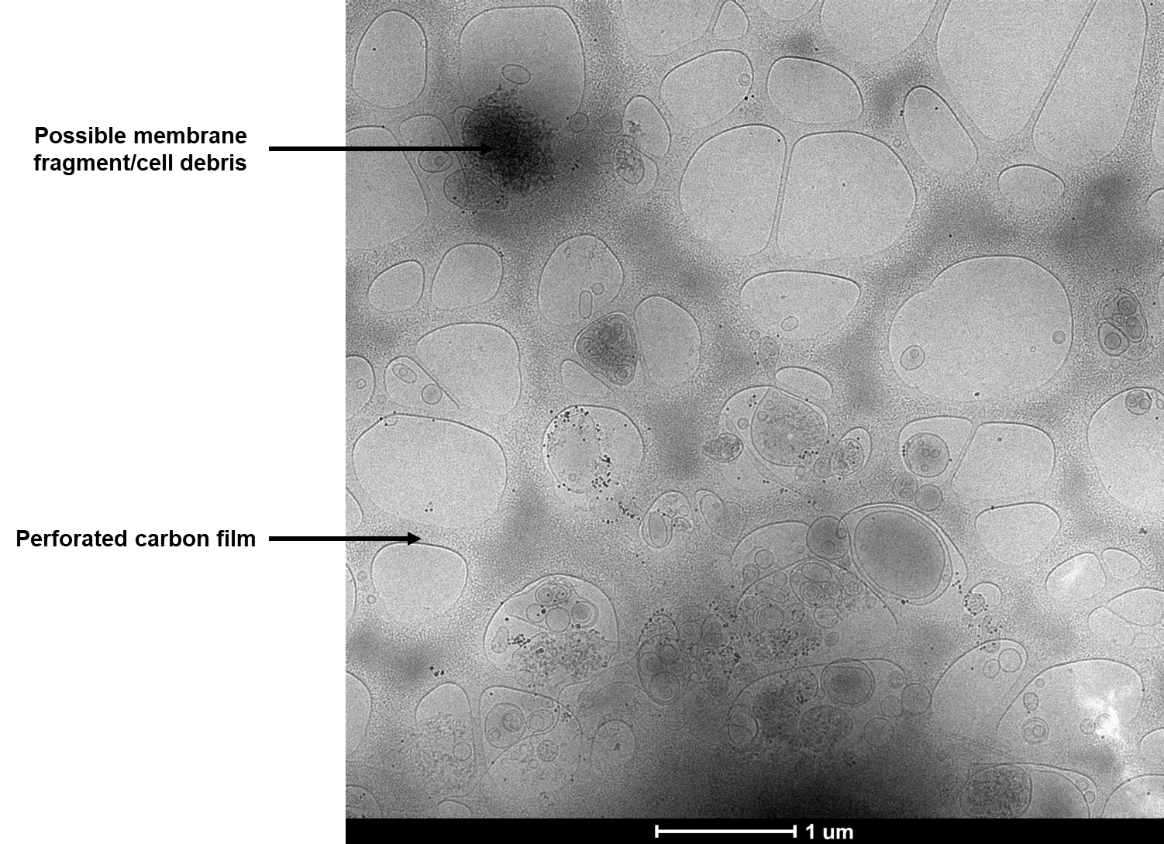


**Figure S1: Cryo-EM Large-Scale Image**

Representative large-scale image captured through cryo-EM of MVs sample. Quantification of contaminants reveals an average of approximately 2 debris spots per image (1.97 ± 0.23) across three replicates (N=3).

**
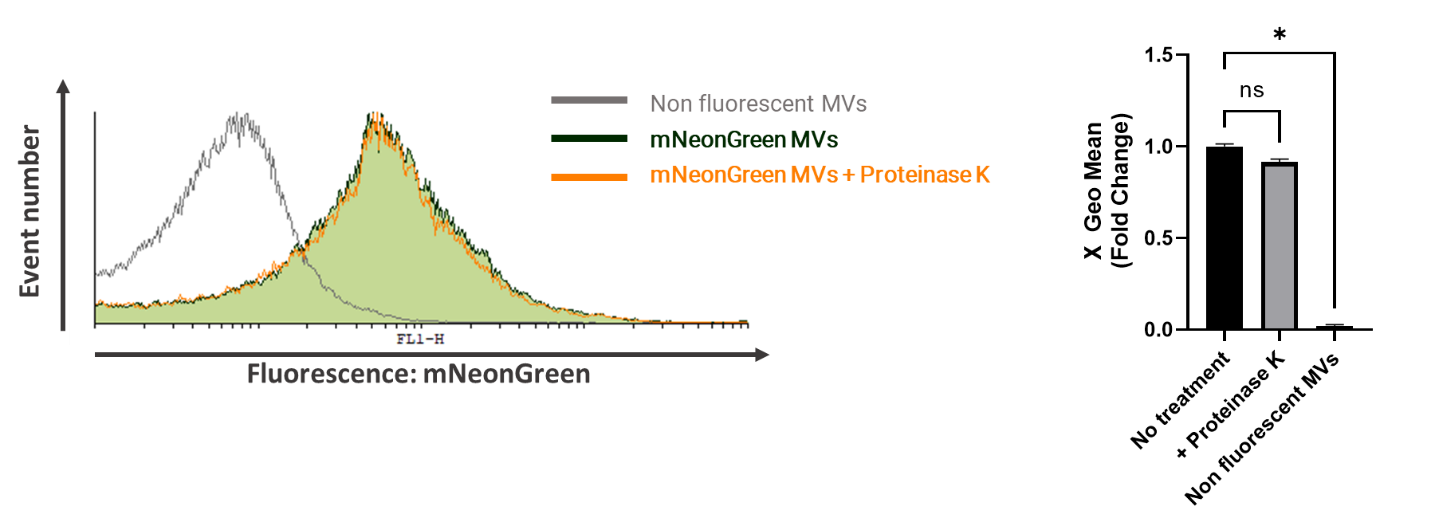
**

**Figure S2: Proteinase K Treatment of MVs - mNeonGreen Encapsulation**

This figure illustrates the outcome of Proteinase K treatment on MVs, assessed through flow cytometry. Despite exposure to Proteinase K, the fluorescence of MVs remained unaltered, indicating robust encapsulation of fluorescence within MVs. This consistent fluorescence profile strongly suggests that the fluorescence is encapsulated within MVs and not associated with the external membrane of MVs. (N=1, n=3).
